# Retracted: Risk Factors of Periodontal Disease: Review of the Literature

**DOI:** 10.1155/2021/8735071

**Published:** 2021-02-12

**Authors:** International Journal of Dentistry

*International Journal of Dentistry* has retracted the article titled “Risk Factors of Periodontal Disease: Review of the Literature” [1]. Since the publication of the article, a significant amount of overlap has been identified with existing publications. The significant sources are listed in the references as [2–5].

The author was asked for clarification but did not provide a response. It is therefore being retracted with the agreement of the editorial board.
